# Supplementary material for: Quantifying Spatiotemporal Gait Parameters with HoloLens in Healthy Adults and People with Parkinson’s Disease: Test-Retest Reliability, Concurrent Validity, and Face Validity
Source: Sensors (Basel). 2020 Jun 5;20(11):3216. doi: 10.3390/s20113216 (PMC7313704; doi:10.3390/s20113216)
Supplement: Supplementary file 1 [file sensors-20-03216-s001.zip › Data/READ ME.pdf]

# Raw and preprocessed time series of the Interactive Walkway and HoloLens

*Supplementary material to: Daphne J. Geerse, Bert Coolen, and Melvyn Roerdink. Quantifying spatiotemporal gait parameters with HoloLens in healthy adults and people with Parkinson's disease: test-retest reliability, concurrent validity and face validity. Sensors.*

---------------------------------

GENERAL INFORMATION

---------------------------------

Daphne J. Geerse
E-mail: d.j.geerse@vu.nl

Bert Coolen
E-mail: h.coolen@vu.nl

Melvyn Roerdink
E-mail: m.roerdink@vu.nl

Department of Human Movement Sciences
Faculty of Behavioural and Movement Sciences
Vrije Universiteit Amsterdam
Amsterdam Movement Sciences
Van der Boechorststraat 7
1081 BT Amsterdam

-----------------------

DATA OVERVIEW

-----------------------

Participants:

- HYA_.. = healthy young adult (n = 22)
- PD_.. = person with Parkinson's disease (n = 23)

Conditions:

- SWS1/SWS2 = slow walking speed trial 1/2
- CWS1/CWS2 = comfortable walking speed trial 1/2

- FWS1/FWS2 = fast walking speed trial 1/2

People with Parkinson's disease only performed comfortable walking speed trials.

Every trial consists of three files (see below for detailed descriptions):

- Preprocessed data as used in the study (Example filename: HYA_01_SWS1.csv)
- Raw Interactive Walkway data (Example filename: HYA_01_SWS1_IWW_raw.csv)
- Raw HoloLens data (Example filename: HYA_01_SWS1_HoloLens_raw.csv)

-----------------------------

PREPROCESSED DATA

-----------------------------

Example filename: HYA_01_SWS1.csv

Preprocessed body points' time series of the Interactive Walkway and time series of the position of the HoloLens in the x-, y- and z-direction. Time series presented are temporally and spatially aligned between systems and preprocessed as detailed in the main text. Body points' time series of the Interactive Walkway are interpolated with a spline algorithm.

The columns represent:

1) SpineBase (x)
2) AnkleLeft (x)
3) AnkleRight (x)
4) SpineShoulder (x)
5) SpineBase (y)
6) AnkleLeft (y)
7) AnkleRight (y)
8) SpineShoulder (y)
9) SpineBase (z)
10) AnkleLeft (z)
11) AnkleRight (z)
12) SpineShoulder (z)
13) HoloLens position (x)
14) HoloLens position (y)
15) HoloLens position (z)

x = anterior-posterior direction (values increase from start to finish on the walkway)

y = vertical direction (values increase from floor to ceiling)

z = mediolateral direction (values increase from the left border to the right border on the walkway)

--------------------------------------------

RAW INTERACTIVE WALKWAY DATA

--------------------------------------------

Example filename: HYA_01_SWS1_IWW_raw.csv

'Raw' time series of 19 body points of the Interactive Walkway in the x-, y- and z-direction. Time series are preprocessed as detailed in the main text, but body points' time series are not interpolated with a spline algorithm.

The columns represent:

| | | |
|---|---|---|
| 1) SpineBase (x) | 20) SpineBase (y) | 39) SpineBase (z) |
| 2) Head (x) | 21) Head (y) | 40) Head (z) |
| 3) ShoulderLeft (x) | 22) ShoulderLeft (y) | 41) ShoulderLeft (z) |
| 4) ElbowLeft (x) | 23) ElbowLeft (y) | 42) ElbowLeft (z) |
| 5) WristLeft (x) | 24) WristLeft (y) | 43) WristLeft (z) |
| 6) HandLeft (x) | 25) HandLeft (y) | 44) HandLeft (z) |
| 7) ShoulderRight (x) | 26) ShoulderRight (y) | 45) ShoulderRight (z) |
| 8) ElbowRight (x) | 27) ElbowRight (y) | 46) ElbowRight (z) |
| 9) WristRight (x) | 28) WristRight (y) | 47) WristRight (z) |
| 10) HandRight (x) | 29) HandRight (y) | 48) HandRight (z) |
| 11) HipLeft (x) | 30) HipLeft (y) | 49) HipLeft (z) |
| 12) KneeLeft (x) | 31) KneeLeft (y) | 50) KneeLeft (z) |
| 13) AnkleLeft (x) | 32) AnkleLeft (y) | 51) AnkleLeft (z) |
| 14) FootLeft (x) | 33) FootLeft (y) | 52) FootLeft (z) |
| 15) HipRight (x) | 34) HipRight (y) | 53) HipRight (z) |
| 16) KneeRight (x) | 35) KneeRight (y) | 54) KneeRight (z) |
| 17) AnkleRight (x) | 36) AnkleRight (y) | 55) AnkleRight (z) |
| 18) FootRight (x) | 37) FootRight (y) | 56) FootRight (z) |
| 19) SpineShoulder (x) | 38) SpineShoulder (y) | 57) SpineShoulder (z) |

x = anterior-posterior direction (values increase from start to finish on the walkway)
y = vertical direction (values increase from floor to ceiling)
z = mediolateral direction (values increase from the left border to the right border on the walkway)

---------------------------

RAW HOLOLENS DATA

---------------------------

Example filename: HYA_01_SWS1_HoloLens_raw.csv

Raw time series the position and orientation of the HoloLens.

The columns represent:

1) Time (in sec)

2) HoloLens position (x)

3) HoloLens position (y)

4) HoloLens position (z)

5) HoloLens orientation (x)

6) HoloLens orientation (y)

7) HoloLens orientation (z)
